# Use of Home-Based Self-Collected Dried Blood Spots to Test for Syphilis, Human Immunodeficiency Virus, Hepatitis C and B Virus Infections and Measuring Creatinine Concentration

**DOI:** 10.1097/OLQ.0000000000001941

**Published:** 2024-01-26

**Authors:** S.A. Nieuwenburg, S.M. Bruisten, T. Heijman, W. Vermeulen, A.P van Dam, M.F. Schim van der Loeff, H.J.C de Vries

**Affiliations:** From the ∗ Department of Infectious Diseases, Public Health Service Amsterdam, Amsterdam, the Netherlands; † Amsterdam Institute for Infection and Immunity; ‡ Department of Medical Microbiology; § Division of Infectious Diseases, Department of Internal Medicine; ¶ Department of Dermatology, Amsterdam UMC, University of Amsterdam, Amsterdam, the Netherlands

## Abstract

**Introduction:**

Home-based self-collected dried blood spot (DBS) sampling could simplify sexual health and preexposure prophylaxis care and reduce sexually transmitted infections (STIs) clinic visits for men who have sex with men (MSM). We compared the performance of DBS to venipuncture collected blood samples to test four STIs and creatinine concentration.

**Methods:**

We invited MSM clients of the Amsterdam STI clinic to participate. Routinely collected peripheral blood was tested for syphilis treponemal antibody, HIV (HIV Ag/Ab), HCV (antibodies), HBV (HBsAg) and creatinine concentration. Participants received a home kit for DBS sampling, a return envelope and a questionnaire to evaluate the acceptability, feasibility and usability of DBS, measured on 5-point Likert scales, 1 representing complete disagreement and 5 complete agreement. We assessed sensitivity and specificity of DBS versus peripheral blood-based testing.

**Results:**

In 2020 to 2021, we included 410 participants; 211 (51.5%) returned a completed DBS card, 117 (28.5%) returned a partially filled card and 82 (20.0%) did not return a card. The sensitivity for syphilis was 90.8% and the specificity 84.3%. For both HIV Ag/Ab and HBsAg, the sensitivity and specificity were 100.0%. The sensitivity for HCV antibody was 80.0%, and the specificity was 99.2%. The DBS creatinine concentration was a mean of 5.3 μmol/L higher than in venipuncture obtained plasma. Participants' median willingness to take a future DBS was 4 (interquartile range, 3–5).

**Discussion:**

Dried blood spot may be an acceptable method among MSM for STI testing and creatinine follow-up during preexposure prophylaxis use. However, collecting enough blood on DBS cards was a challenge, and sensitivities for syphilis and HCV serology were too low.

## Abstract 2

Testing for sexually transmitted infections in home-based self-collected dried blood spots was not sensitive and specific enough and collecting enough blood on filter cards was a challenge.

Centres for Sexual Health (CSH) guidelines recommend regular testing for sexually transmitted infections (STIs) in men who have sex with men (MSM), especially when preexposure prophylaxis (PrEP) for HIV is used.^1^ Depending on the risk, MSM on PrEP may be regularly tested for serum creatinine concentration. In practice, MSM are often tested less frequently than desired due to clinic capacity constraints or personal circumstances.^2^ Centres for Sexual Health in the Netherlands provide free-of-charge testing of MSM for HIV, hepatitis B virus (HBV), syphilis, *Chlamydia trachomatis* and *Neisseria gonorrhoeae*, and for those on PrEP measurement of plasma creatinine if indicated. Testing for other STIs may also be performed if indicated, such as for genital herpes, lymphogranuloma venereum, mpox, and hepatitis C virus (HCV). The CSH also provides STI treatment and partner notification, HIV care referral to specialized clinics, PrEP care, and sexual health counseling.

To increase and improve STI testing among key populations, various outreach interventions have been proposed, including web-based strategies combined with home-based self-sampling.^3^ Self-sampling strategies have proven to be of added value in STI control.^4,5^ These strategies could be especially helpful for people belonging to key populations that do not visit a CSH or other health care facilities.

In the Netherlands, most self-sampling strategies for STI control focus on testing for *C. trachomatis* and *N. gonorrhoeae* using self-taken swabs or urine samples. Yet, a health care provider is needed to collect venous blood samples for screening of infections like syphilis HIV, HCV, and HBV. Also, PrEP use requires regular renal function screening with plasma creatinine concentration measurement. Home-based self-sampling followed by sending the collected samples via surface mail to the laboratory for STI and serum creatinine testing could decrease the burden for the client, as well as for the clinic.^6^ The capacity of the CSH in Amsterdam is limited so testing policy could be streamlined by offering self-sampling to a proportion of the clients.

Via a finger prick and using filter paper, whole blood samples can be self-collected safely. Blood droplets are soaked by the filter paper and after drying sent easily by surface mail for further processing and testing in a laboratory. Studies have been conducted into the test characteristics of fourth-generation HIV screening tests on home-based self-collected dried blood spot (DBS) sampling versus venous blood samples.^7,8^ Several studies suggested that combined screening for syphilis, HIV, HCV, and HBV on DBS may be as reliable as screening based on venous blood samples.^9–14^

Earlier studies showed the feasibility and reliability of assessing creatinine concentrations based on DBS, such studies were done in nephrology patients,^15,16^ but not yet in clients of STI clinics eligible for HIV PrEP.

In this study, we evaluated the performance of self-collected DBS for serological screening of syphilis, HIV, HCV, and HBV and creatinine measurement among MSM compared with routine testing performed on health provider collected peripheral blood samples via venipuncture during a consultation at the CSH in Amsterdam. Moreover, we evaluated the acceptability, feasibility, and usability of self-collected DBS.

## Methods

### Study Design and Population

The CSH of the Amsterdam Public Health Service, the Netherlands, is a STI clinic performing approximately 50,000 consultations annually. Consultations are at the client's own initiative, anonymous and free-of-charge. We invited MSM clients 18 years and older to participate in the study. Our goal was to include 200 participants with completely filled DBS cards.

During consultation, eligible clients were asked to participate in the study. Upon consent, two blood samples (serum and ethylenediaminetetraacetic acid [EDTA]) were taken by a health provider. Thereafter participants were given a home kit with 2 lancets, including a link to an app with step-by-step instructions (Supplementary Fig. 1, http://links.lww.com/OLQ/B52) and additional short instructions on paper on the collection of blood on the 5 spots of the DBS filter paper cards (Whatman 903 protein saver cards). Participants were asked to fill all 5 spots of the DBS card at home within 24 hours of the consultation at the CSH, and to complete a questionnaire. The questionnaire included statements on the instructions, experiences of self-sampling, preference for settings (home vs CSH), and future use of DBS using 5-point Likert scales, where 1 represents complete disagreement and 5 represents complete disagreement with the statements. The DBS card and questionnaire were then returned by surface mail in a preaddressed envelope suitable for clinical specimens to the Amsterdam Public Health Laboratory (PHL). Upon returning the DBS card and questionnaire, participants were rewarded with a gift card of 20 euro as a token of appreciation for their participation

### Serum-, EDTA Blood Tube, and DBS Work-Up

At the Amsterdam PHL, the serum tube was immediately processed and used for routine testing for syphilis, HIV, HCV, and HBV. For possible follow-up tests, serum samples were stored at −20°C. The EDTA plasma sample was aliquoted in microtubes and stored at −20°C. For the DBS card, the arrival date was noted at the PHL. The quality of the spots was assessed visually by a laboratory technician to determine whether the spots were fully saturated and one spot was removed from the card and stored at −20°C. The time of testing differed per STI but in each case, this was performed batch wise. As soon as samples were eluated, they were tested immediately for all microbiological antibody tests. From 190 clients, the DBS spot together with an EDTA aliquot was sent to the Pharmacy Laboratory of the Amsterdam UMC, location AMC, at the end of the study for batchwise analysis of creatinine levels.

The four remaining spots were stored at 4°C to 8°C until batch wise analysis was performed by punching 5-mm discs (Kangaro, Zuthpen, the Netherlands) which were placed in a microtube. Per disc 250-μL PBS with 0.05% tween buffer was used to elute the blood by mildly shaking for 3 hours. After elution and centrifugation to remove red blood cells, the eluate was used for the screenings tests. If a serum sample or DBS card had insufficient volume, as judged visually, the following order of testing was used: (1) syphilis, (2) HIV, (3) (HCV), (4) HBV, and (5) creatinine.

### Diagnostic Tests on Serum, Plasma, and DBS

At the PHL, testing for syphilis, HIV Ag/Ab, HCV, HBsAg are all performed on the Liaison XL Murex (Diasorin, Italy). Screening for syphilis is done with a treponemal test using chemiluminescence technology (CLIA). In routine clinic practice, confirmation tests are done for all infections, but such testing was not evaluated in this study.

Measurement of creatinine concentration (expressed in *μmol*/*L*) was performed at the Pharmacy Laboratory of the Amsterdam UMC, location AMC. The samples were analyzed by liquid chromatography with tandem mass spectrometry (LC/MS) (Shimadzu, Kyoto, Japan; SCIEX, Concord, Canada).

### Sample Size

This study was powered for the evaluation of syphilis and HIV testing on DBS. The positivity rate of CLIA and the HIV prevalence among MSM visiting the CSH in Amsterdam in 2019 was used to calculate the sample size. The positivity rate of CLIA was 30.0% and the prevalence of known HIV-infections among MSM visiting the CSH was 16.0%. With 200 participants and an expected CLIA positivity rate of 30.0% and an expected sensitivity of the DBS methods of 100.0%, the expected precision (95% confidence interval [CI]) was 94.0% to 100.0%). Assuming a specificity of 99.0%, the expected precision around that estimate was 96.1% to 100.0%. With 200 participants and an expected HIV prevalence of 16.0% and an expected sensitivity of the DBS method of 100.0%, the expected precision was (95% CI) 89.1% to 100.0%. If specificity was 99.0%, the expected precision around that estimate was 95.8% to 99.9%. During the study, it became clear that a substantial proportion of participants did not return completed DBS cards or sent incompletely filled cards. We continued recruitment until 200 fully completed DBS cards had been received.

### Statistical Analysis

Categorical variables were described using count data and proportions. Continuous variables were described using median and interquartile range (IQR). The results of the DBS were compared with the results of the routine tests as performed on the provider-collected peripheral blood sample. For syphilis, HIV, HCV, and HBV, the sensitivity and specificity with 95% CI of the performance of self-collected DBS compared with provider collected serum samples were assessed. The level of agreement between plasma creatinine and DBS creatinine was analyzed with a Passing-Bablok regression (a robust, nonparametric statistical procedure that enables estimation of agreement between analytical methods and detection of possible systematic bias between them) and a Bland-Altman plot.^17,18^ The items of the questionnaire were described using median and interquartile range (IQR). Scores 4 and 5 were considered as agreement with a statement. We estimated odds ratios (ORs) and their 95% CI using univariable and multivariable logistic regressions to assess associations between determinants and agreeing with the statements in the questionnaire. We carried out analyses using Stata (v15.1, StataCorp, College Station, TX). Level of significance was set at <0.05.

### Ethics Statement

A positive screening in DBS in combination with a negative routine screening test was not followed up since test results based on the provider-collected peripheral blood samples were considered standard of care. This study was approved by the Medical Ethics Committee of the Amsterdam University Medical Centers (NL68213.018.18, 2018_302).

## Results

**Figure 1:**
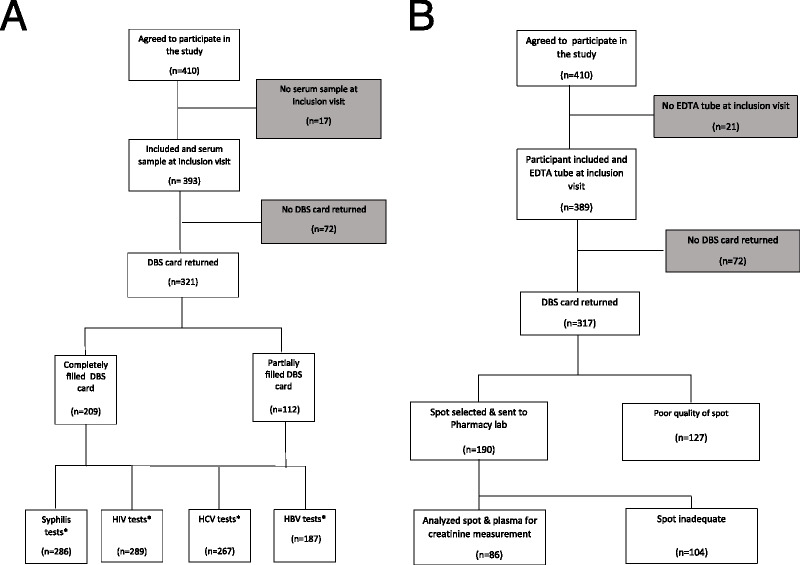
A, Flowchart of clients in the study on dried blood spot screening at the Centre for Sexual Health from November 2020 to October 2021 regarding serum samples for the screening of syphilis, HIV HCV, and HBV, Amsterdam, the Netherlands. B, Flowchart of clients in the study at the Centre for Sexual Health from November 2020 to October 2021 regarding plasma samples for creatinine concentration measurement, Amsterdam, the Netherlands. *During the study the serum sample in some cases was lost due to human/technical error or there was visually insufficient blood on the spots for the screening tests.

From November 2020 through October 2021, 410 MSM visiting the CSH Amsterdam agreed to participate in the study (Fig. 1). The median age was 36 years (IQR, 29–47 years) (Table 1). Forty-nine (12.0%) participants were known to be living with HIV and of those 47 (95.9%) were on antiretroviral therapy. A serum sample was available for 393 (95.9%) participants and an EDTA sample for 389 (94.9%). In total, 328 clients (80%) returned a DBS card; in 211 cards all 5 spots were filled, and 117 cards were not completely filled (Fig. 1).

**TABLE 1:** Socio-Demographics, Sexual Behavior, Health Status and Sample Availability of 410 MSM Clients Agreeing to Participate in Study on Dried Blood Spot Testing, November 2020 to October 2021, at the Centre for Sexual Health Amsterdam, the Netherlands

	Total, N = 410	
	n*	%*
Age, y		
Median [IQR]	36	[29–47]
<35	191	46.6%
35–54	173	42.2%
≥55	46	11.2%
Country of origin		
The Netherlands	235	57.3%
Other	175	42.7%
Education		
None/primary/secondary school	66	16.1%
College/university	325	79.3%
Unknown	19	4.6%
Sexual behavior^†^		
MSM	404	98.5%
MSMW	6	1.5%
HIV status before consultation		
Positive	49	12.0%
Negative	358	87.3%
Never tested	3	0.7%
cART use^‡^		
No	2	4.1%
Yes	47	95.9%
Most recent CD4 count (cells/μL)		
<350	1	2.0%
350–499	6	12.2%
≥500	28	57.1%
Unknown	14	28.6%
Antibiotic use^§^		
No	360	87.8%
Yes	48	11.7%
Unknown	2	0.5%
Availability of serum sample		
No	17	4.1%
Yes	393	95.9%
Availability of EDTA sample		
No	21	5.1%
Yes	389	94.9%
Status of DBS cards		
Completely filled	211	51.5%
Partially filled	117	28.5%
Unknown/not returned	82	20.0%

* Unless otherwise stated. ^†^In the 6 months before the consultation. ^‡^In patients with HIV. ^§^In the previous 3 months. MSMW, men who have sex with men and women; cART, combination antiretroviral therapy.

We evaluated the test performance of DBS-based testing compared with routine diagnostics based on serum obtained through venipuncture (Table 2 and Supplementary Table 1, http://links.lww.com/OLQ/B52). Because of incompletely filled cards, numbers differ between performed STI tests. There were 120/286 (42.0%) clients positive for syphilis CLIA with the test performed on serum obtained by venipuncture. With the test performed on DBS collected blood, there were 11 false-negative and 26 false-positive results; sensitivity was 90.8% (95% CI, 84.2–95.3%) and specificity 84.3% (95% CI, 77.9–89.5%). There were 27/289 (9.3%) clients positive for HIV Ag/Ab when performed on serum obtained by venipuncture. The test performed on DBS collected blood had a sensitivity of 100.0% (95% CI, 87.2–100.0%) with a specificity of 100.0% (95% CI, 98.6–100.0%). There were 10/267 (3.7%) samples positive for HCV antibodies in testing performed on serum obtained by venipuncture. With the test performed on DBS collected blood, there were 2 false-negative and 2 false-positive results, thus sensitivity was 80.0% (95% CI, 44.4–97.5%) and specificity was 99.2% (95% CI, 97.2–100.0%). Only 2 of 187 (1.1%) clients were positive for HBsAg in testing performed on serum obtained by venipuncture. Sensitivity of tests performed on DBS collected blood was 100.0% (95% CI, 15.8–100.0%) and specificity was also 100.0% (98.0–100.0%).

**TABLE 2:** Comparison of Screenings Tests Performed on Client Home Collected DBS Versus Provider Clinic Collected Serum Samples, in Participants Visiting the Centre for Sexual Health Amsterdam, November 2020 to October 2021

Test	Pairs of Serum Samples and DBS Tested (N)	No. Samples Serum/DBS*				Sensitivity (95% CI)	Specificity (95% CI)
		+/+	+/−	−/+	−/−		
Syphilis CLIA	286	109	11	26	140	90.8% (84.2–95.3%)	84.3% (77.9–89.5%)
HIV Ag/Ab	289	27	0	0	262	100.0% (87.2–100.0%)	100.0% (98.6–100.0%)
HCV antibody	267	8	2	2	255	80.0% (44.4–97.5%)	99.2% (97.2–99.9%)
*HBsAg*	187	2	0	0	185	100.0% (15.8–100.0%)	100.0% (98.0–100.0%)

*Numbers in these four columns refer to numbers of paired samples that have the indicated combination of test results from serum and DBS based testing. N, number.

**Figure 2:**
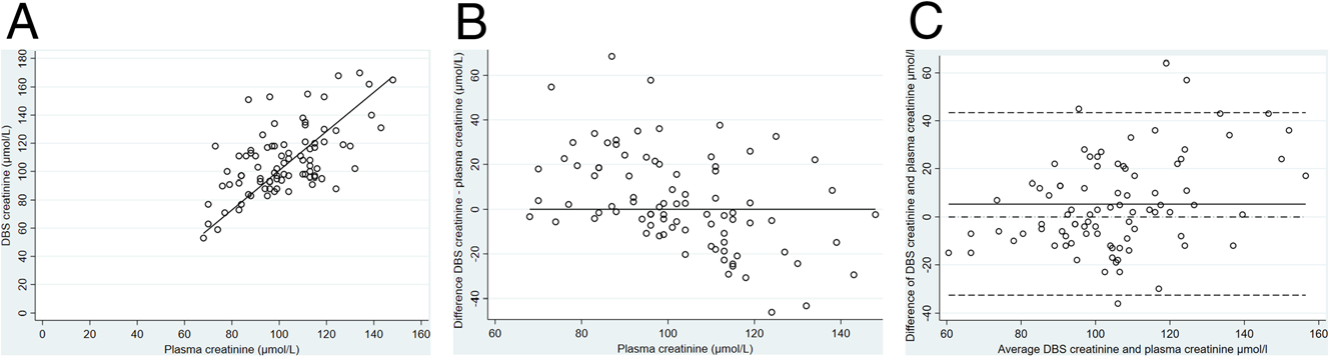
Passing and Bablok regression analyses of comparison between plasma creatinine and DBS creatinine levels (N = 86). (A) Scatter diagram with a continuous line. This line is the Passing & Bablok regression line *y* = −38.1*x* − 1.4 (95% CI slope, −68.0 to −10.2; intercept, 1.1 to 1.70). Cusum test for linearity indicates no significant deviation from linearity (*P* > 0.20). (B) Residual plot presents distribution of difference around fitted regression line. (C) The panel shows the Bland-Altman analysis with a bias of 5.29 μmol/L (95% CI, 1.19 to 9.51) shown by the continuous line; the dashed lines indicate 95% limits of agreement.

More than half of DBS (104/190 [55.0%]) samples did not meet the quality standards of the Pharmacy laboratory of the Amsterdam UMC required for creatinine concentration analysis, mostly because of insufficient blood volumes in DBS. For only 86 of 190 (45.0%) cases paired DBS and plasma creatinine samples could be analyzed. The mean plasma creatinine value was 101.4 μmol/L (SD, 16.4); mean creatinine concentration in DBS-based samples was 107.8 (SD, 24.1) (Fig. 2). The mean difference was 5.3 μmol/L (95% CI, 1.2–9.5). The DBS creatinine concentrations were on average 5.9% higher than the plasma samples for creatinine.

A total of 317 of 410 (77.3%) participants completed the questionnaire on the acceptability, feasibility, and usability of self-collected DBS. Participants agreed that the instructions were clear with a median score of 4 (IQR, 4–5), and that it was easy to do a finger prick with a median score of 4 (IQR, 3–5) (Table 3). However, applying the blood onto the spot card was reported to be more challenging with a median score of 3 (IQR, 2–4). Respondents were, on average, neutral to a preference of DBS at home rather than coming to a clinic with a median score of 3 (IQR, 2–4). Participants indicated that they would do a blood spot test again in the future with a median score of 4 (IQR, 3–5).

**TABLE 3:** The Acceptability, Feasibility, and Usability of DBS Testing at Home in Study Participants, Centre for Sexual Health Amsterdam, Between November 2020 and October 2021 (N = 317)

Statements*	Median [IQR]
Experience of use	
(1) The instructions on how to do a finger-prick were clear	4 [4–5]
(2) It was easy to do the finger-prick	4 [3–5]
(3) It is unpleasant to do the finger-prick	2 [2–3]
(4) To apply the blood on the cards is easy	3 [2–4]
Comparing care settings	
(5) I prefer a finger-prick over blood drawing by a clinic professional	3 [2–4]
(6) I value a consultation with a nurse/doctor more than the ability to do a self-test at home	3 [2–4]
(7) I prefer to do the bloodspot test at home rather than come to the STI clinic	3 [2–4]
Future use	
(8) I would do the bloodspot test again	4 [3–5]

* Statements were scored on a Likert scale 1 to 5 (completely disagree to completely agree). Data provided for those who completed all items in the whole questionnaire.

We assessed the determinants of agreeing (4 or 5 on Likert scale) with each statement. In the univariable model, regarding question 7 “I prefer to do the bloodspot test at home rather than come to the STI clinic,” those in the age category 35–54 years were more likely to prefer the DBS test than those younger than 35 years (OR, 1.5; 95% CI, 0.9–2.4; Supplementary Table 2, http://links.lww.com/OLQ/B52), and those in the age category 55 years and above were less likely to prefer a DBS rather than a test at the CSH (OR, 0.4; 95% CI, 0.2–1.0). In multivariable models, the only statistically significant association was that those born outside the Netherlands more often preferred a consultation with a nurse/doctor over a DBS (OR, 1.7; 95% CI, 1.1–2.8).

## DISCUSSION

In this study, we evaluated the use of dried blood spot self-sampling at home as an alternative to blood samples collected by venipuncture in the clinic for syphilis, HIV, HCV, and HBV testing and creatinine concentration measurement in MSM. For HIV Ag/Ab and HBsAg, the sensitivity and specificity were both 100.0%, although with a wide 95% CI for HBsAg. For syphilis CLIA and HCV antibody test, we found sensitivities that are too low for routine diagnostic purposes. Regarding the creatinine concentration determination, more than half of the spots could not be evaluated due to low volumes or low quality of the sample. The successfully analyzed DBS samples showed on average a slightly higher creatinine concentration (5.9%) compared with plasma samples. The study population appreciated DBS as an acceptable sampling method and most expressed willingness to perform DBS self-sampling again in the future. These findings support the acceptability of DBS self-sampling in MSM. However the feasibility of using self-sampling for STI testing through DBS is not supported with only 211 of 410 participants (51%) having all 5 spots filled for testing.

Although participants stated that the instructions on how to perform a finger-prick test were clear (median score, 4; IQR, 4–5), it was observed that only 51.0% of the returned spot cards had sufficient blood volumes in five visually identifiable spots with good quality. This illustrates the challenges that participants may have had with collecting enough blood and properly applying blood droplets onto the spot cards. Possibly, the type of lancet may have played a role in this; the lancet's design and depth of incision may impact the ease and effectiveness of blood collection.

For syphilis (CLIA), the sensitivity of DBS-based testing was 90.8% and the specificity was 84.3%. In a study from Tanzania, over 1600 paired samples were tested with several commercially available diagnostic syphilis assays. Results obtained using DBS were compared with those obtained using plasma samples as a reference.^19^ The treponemal test used on DBS found varying results depending on the treponemal test used. Treponema pallidum hemagglutination assay (sensitivity 50.5%) and EIA (specificity 50.4%) showed poor performance, whereas TPPA showed a high sensitivity (95.5%) and specificity (99%).^19^ Similar results were seen in other studies using TPPA.^13,14^

We found a low sensitivity (80.0%) to detect HCV antibodies, whereas other studies reported a much higher sensitivity (>97%) and comparable specificity (99%).^11,14,20^ The number (n = 10) of anti-HCV positive participants included in our study was low. The sensitivity and specificity for both HIV Ag/Ab and HBsAg were both 100.0%, but the 95% confidence limits for HBsAg were wide due to its low prevalence (1.1%). Our findings on HIV and HBV align with those of previous studies.^9,13,14^

Several studies on DBS-based measuring of creatinine measurements have been done in patients (mainly nephrology patients), with good results.^16,21–23^ DBS creatinine in nephrology patients was thought to be feasible and could reduce patient burden in monitoring graft function compared with the conventional venous blood sampling. Similar studies in healthy individuals are rare.^24^

To the best of our knowledge, there have been no other published reports comparing the performance of these five tests (syphilis, HIV, HCV, HBV, and creatinine) on DBS samples specifically in the MSM population, a key population for these infections and for PrEP indications requiring creatinine monitoring. The combination of these tests together with the previously evaluated self-sampling for *N. gonorrhoeae* and *C. trachomatis,*^25–27^ may help to offer individuals a comprehensive STI home kit but the issue of insufficient blood on the cards should be resolved. This kit would be particularly beneficial for individuals who are currently not attending health care institutes for testing (hidden population). In such cases, the lower sensitivities of syphilis and HCV might be deemed acceptable for the convenience and accessibility that home-based testing provides.

Our study had several limitations. The preanalytic manual steps of punching, transfer, and elution are time consuming steps, prone to errors. This makes it difficult to integrate this test method in a regular medical laboratory. This work-up cannot easily be automated, which would be preferred, especially when dealing with a large number of samples. This manual labor also means that the DBS method will be very costly. Second, as the sampling was performed at home, we do not know the volume of blood applied onto the DBS cards and how participants applied the blood to the cards. The main problem with the partially filled spots was that blood samples were too small and thus the blood volume was insufficient to perform all planned tests. The partially filled spots may be due to instructions that were not clear enough. Assessment of adequate filling of spots was not straightforward. Many spots, initially assessed as adequate by the PHL, were rejected as insufficient by the Pharmacy Lab at Amsterdam. Third, willingness to participate in the study may have been affected by the COVID restrictions, as the STI clinic became less accessible. Furthermore, we do not know the reasons for not completing the cards or the questionnaire for those who did not submit either; this may have influenced our evaluation of the acceptability, feasibility, and usability of the DBS. Last, for those with a prior history of syphilis a treponemal screening test is not indicated since treponemal antibodies persist after adequate therapy, and a nontreponemal test, such as rapid plasma reagin, is needed to distinguish an adequately treated infection from a repeat infection. Yet, so far nontreponemal tests cannot be performed on DBS samples as they require fresh blood for analysis. This means that clients with a history of syphilis cannot be screened for repeat infections with DBS and samples collected through venipuncture will still be needed.

The main benefit of the DBS method is that it can be performed through self-sampling at home. Another benefit is that the samples are very stable (do not disintegrate) during transportation and storage, as long as they are dried well. Although participants reported that instructions were clear, we recommend step-by-step visual instructions or a short film with instructions and, if possible, to combine this with performing the spots for a first time with a clinician, so that technical and logistical challenges and questions can be addressed. This may be helpful in improving the collection of adequate volumes. For those who do not return the kit a text message reminder could be sent, because previous studies have shown increased sample kit-return with reminders.^6,28^

In conclusion, DBS may be an acceptable self-sampling method among MSM for the screening of sexually transmitted infections, but obtaining enough blood on the spots for a complete STI work-up is a challenge, and observed sensitivities and specificities for 2 of the 4 indicated infections were too low. The findings derived from our study can be used to optimize and facilitate the implementation of home based collections for STI testing. Furthermore further research is needed to develop a testing strategy of non-treponemal test on DBS samples.

## Supplementary Material

SUPPLEMENTARY MATERIAL
