# Supplementary material for: Use of Home-Based Self-Collected Dried Blood Spots to Test for Syphilis, Human Immunodeficiency Virus, Hepatitis C and B Virus Infections and Measuring Creatinine Concentration
Source: Sex Transm Dis. 2024 Jan 26;51(4):283–8. doi: 10.1097/OLQ.0000000000001941 (PMC11520340; doi:10.1097/OLQ.0000000000001941)
Supplement: SUPPLEMENTARY MATERIAL [file std-51-283-s001.docx]

**Supplementary File**

**Use of home-based self-collected dried blood spots to test for syphilis, HIV, hepatitis C and B virus infections and measuring creatinine concentration.**

S.A. Nieuwenburg^1,2^ MD, S.M. Bruisten^1,2^ PhD, T. Heijman^1^ PhD, W. Vermeulen^1^ BSc, A.P van Dam^1,3^ MD PhD, M.F. Schim van der Loeff^1,2,4*^ MD PhD, H.J.C de Vries^1,2,5*^ MD PhD.

^1^ Department of Infectious Diseases, Public Health Service Amsterdam, Amsterdam, the Netherlands

^2^ Amsterdam UMC location University of Amsterdam, Amsterdam institute for Infection and Immunity, Meibergdreef 9, Amsterdam, the Netherlands

^3^ Amsterdam UMC location University of Amsterdam, Department of Medical Microbiology, Meibergdreef 9, Amsterdam, the Netherlands

^4^ Amsterdam UMC location, University of Amsterdam, Department of Internal Medicine, Division of Infectious Diseases, Meibergdreef 9, Amsterdam, the Netherlands

^5^ Amsterdam UMC location University of Amsterdam, Department of Dermatology, Meibergdreef 9, Amsterdam, the Netherlands

** Shared last author*

Supplementary Figure 1. **Screenshots of step-by-step instruction of dried blood spot collection**


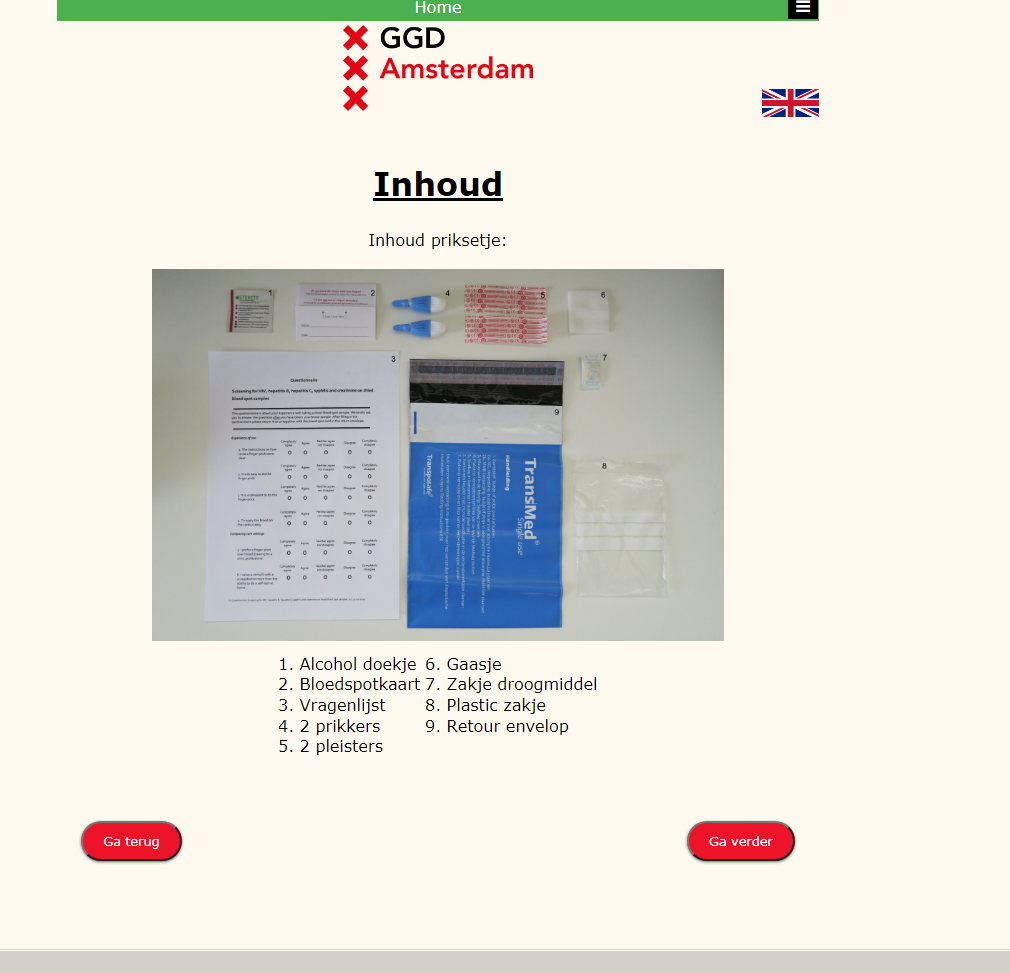

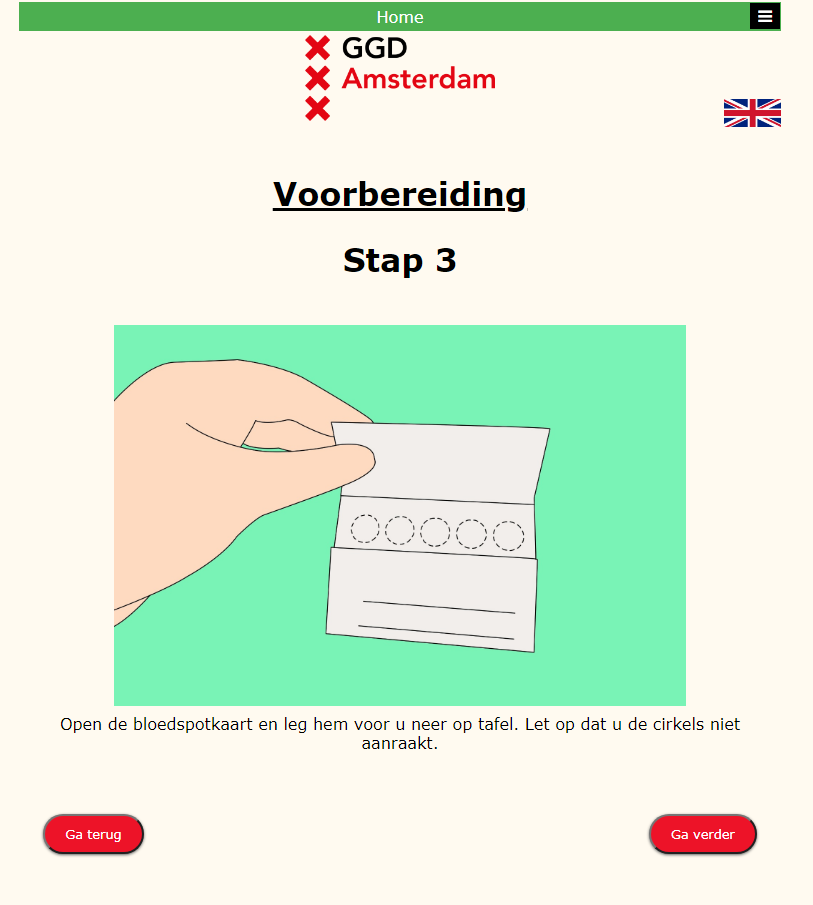
* Text upper screenshot reads: content of the needle set: 1. Alcohol swab, 2. Blood spot card, 3. Questionnaire, 4. 2 lancets, 5. 2 plasters, 6. Gauze, 7. Desiccant packet. 8. Plastic bag, 9. Return envelope.

*Test lower screenshot reads: preparation step 3. Open the blood spot card and place it in front of you on the table. Be careful not to touch the circles.

Supplementary Table 1a **2x2 table of comparison of screenings test of syphilis CLIA performed on client home collected DBS versus provider clinic collected serum samples, in participants visiting the Centre for Sexual Health Amsterdam, November 2020 - October 2021.**

|  | Serum samples | |  |
| --- | --- | --- | --- |
| DBS samples | Positive | Negative | Total |
| Positive | 109 | 26 | 135 |
| Negative | 11 | 140 | 151 |
| Total | 120 | 166 | 286 |

CLIA = chemiluminescent immunoassay

Supplementary Table 1b **2x2 table of comparison of screenings test of HIV Ag/Ab performed on client home collected DBS versus provider clinic collected serum samples, in participants visiting the Centre for Sexual Health Amsterdam, November 2020 - October 2021.**

|  | Serum samples | |  |
| --- | --- | --- | --- |
| DBS samples | Positive | Negative | Total |
| Positive | 27 | 0 | 27 |
| Negative | 0 | 262 | 262 |
| Total | 27 | 262 | 289 |

Ag = antigen; Ab = antibody

Supplementary Table 1c **2x2 table of comparison of screenings test of HCV antibody performed on client home collected DBS versus provider clinic collected serum samples, in participants visiting the Centre for Sexual Health Amsterdam, November 2020 - October 2021.**

|  | Serum samples | |  |
| --- | --- | --- | --- |
| DBS samples | Positive | Negative | Total |
| Positive | 8 | 2 | 10 |
| Negative | 2 | 255 | 257 |
| Total | 10 | 257 | 267 |

HCV = hepatitis C virus

Supplementary Table 1d **2x2 table of comparison of screenings test of HBV** **HBsAg performed on client home collected DBS versus provider clinic collected serum samples, in participants visiting the Centre for Sexual Health Amsterdam, November 2020 - October 2021.**

|  | Serum samples | |  |
| --- | --- | --- | --- |
| DBS samples | Positive | Negative | Total |
| Positive | 2 | 0 | 2 |
| Negative | 0 | 185 | 185 |
| Total | 2 | 185 | 187 |

HBV = hepatitis B virus; HBsAg = Hepatitis B virus surface Antigen;

**Supplementary table 2 Univariable and multivariable analysis of determinants** **on agreeing with statements regarding acceptability, feasibility, and usability of DBS testing in participants completing the questionnaire November 2020 - October 2021, Centre for Sexual Health Amsterdam, the Netherlands.**

|  | **1. Instructions were clear** | | **2. Finger-prick was easy** | | **3. Finger-prick was unpleasant** | | **4. Applying blood on card was easy** | | **5. Preference finger-prick over venous blood test** | | **6. Value consultation at clinic** | | **7. Preference DBS at home over clinic** | | **8. Future use DBS** | |
| --- | --- | --- | --- | --- | --- | --- | --- | --- | --- | --- | --- | --- | --- | --- | --- | --- |
| ***Univariable*** | | | | | | | | | | | | | | | | |
|  | *OR* | *95% CI* | *OR* | *95% CI* | *OR* | *95% CI* | *OR* | *95% CI* | *OR* | *95% CI* | *OR* | *95% CI* | *OR* | *95% CI* | *OR* | *95% CI* |
| **Age** |  |  |  |  |  |  |  |  |  |  |  |  |  |  |  |  |
| <35 years | REF |  | REF |  | REF |  | REF |  | REF |  | REF |  | REF |  | REF |  |
| 35-54 years | 0.80 | 0.43-1.48 | 0.90 | 0.53-1.53 | 0.85 | 0.46-1.55 | 1.55 | 0.95-2.53 | 1.31 | 0.82-2.11 | 0.72 | 0.45-1.17 | 1.47 | 0.92-2.36 | 1.01 | 0.58-1.74 |
| ≥55 years | 0.44 | 0.19-1.02 | 0.62 | 0.29-1.36 | 0.66 | 0.23-1.84 | 1.26 | 0.58-2.71 | 0.61 | 0.27-1.36 | 1.29 | 0.62-2.68 | 0.43 | 0.18-1.00 | 0.51 | 0.23-1.11 |
| **Country of birth** |  |  |  |  |  |  |  |  |  |  |  |  |  |  |  |  |
| The Netherlands | REF |  | REF |  | REF |  | REF |  | REF |  | REF |  | REF |  | REF |  |
| Other | 1.67 | 0.93-3.01 | 1.21 | 0.73-1.99 | 0.82 | 0.45-1.47 | 1.03 | 0.65-1.63 | 0.87 | 0.50-1.38 | 1.50 | 0.95-2.36 | 0.69 | 0.44-1.08 | 1.02 | 0. 62-1.70 |
| **Education level** |  |  |  |  |  |  |  |  |  |  |  |  |  |  |  |  |
| None/primary/  secondary school | REF |  | REF |  | REF |  | REF |  | REF |  | REF |  | REF |  | REF |  |
| College/University | 1.36 | 0.66-2.79 | 1.17 | 0.60-2.28 | 0.78 | 0.37-1.65 | 1.10 | 0.58-2.09 | 1.23 | 0.66-2.31 | 0.70 | 0.38-1.29 | 1.48 | 0.79-2.80 | 1.74 | 0.91-3.32 |
| **HIV status** |  |  |  |  |  |  |  |  |  |  |  |  |  |  |  |  |
| Negative | REF |  | REF |  | REF |  | REF |  | REF |  | REF |  | REF |  | REF |  |
| Positive | 0.55 | 0.24-1.26 | 0.68 | 0.31-1.48 | 0.86 | 0.31-2.34 | 1.33 | 0.63-2.82 | 1.46 | 0.69-3.07 | 1.84 | 0.87-3.88 | 0.63 | 0.29-1.39 | 0.73 | 0.33-1.61 |
| ***Multivariable*** | | | | | | | | | | | | | | | | |
| **Age** |  |  |  |  |  |  |  |  |  |  |  |  |  |  |  |  |
| <35 years | REF |  | REF |  | REF |  | REF |  | REF |  | REF |  | REF |  | REF |  |
| 35-54 years | 0.81 | 0.43-1.52 | 0.81 | 0.47-1.41 | 0.84 | 0.45-1.55 | 1.42 | 0.85-2.36 | 1.30 | 0.79-2.14 | 0.70 | 0.43-1.16 | 1.58 | 0.96-2.59 | 1.02 | 0.58-1.79 |
| ≥55 years | 0.53 | 0.22-1.31 | 0.69 | 0.30-1.63 | 0.58 | 0.20-1.72 | 1.24 | 0.55-2.83 | 0.57 | 0.24-1.35 | 1.23 | 0.55-2.73 | 0.47 | 0.19-1.15 | 0.64 | 0.27-1.48 |
| **Country of birth** |  |  |  |  |  |  |  |  |  |  |  |  |  |  |  |  |
| The Netherlands | REF |  | REF |  | REF |  | REF |  | REF |  | REF |  | REF |  | REF |  |
| Other | 1.44 | 0.78-2.64 | 1.18 | 0.70-2.01 | 0.84 | 0.46-1.53 | 0.97 | 0.60-1.59 | 0.80 | 0.49-1.30 | 1.72 | 1.07-2.78 | 0.63 | 0.39-1.02 | 0.94 | 0.55-1.60 |
| **Education level** |  |  |  |  |  |  |  |  |  |  |  |  |  |  |  |  |
| None/primary/  secondary school | REF |  | REF |  | REF |  | REF |  | REF |  | REF |  | REF |  | REF |  |
| College/University | 1.11 | 0.52-2.38 | 1.06 | 0.53-2.13 | 0.74 | 0.34-1.59 | 1.14 | 0.59-2.23 | 1.19 | 0.62-2.31 | 0.78 | 0.41-1.48 | 1.25 | 0.64-2.44 | 1.58 | 0.80-3.09 |
| **HIV status** |  |  |  |  |  |  |  |  |  |  |  |  |  |  |  |  |
| Negative | REF |  | REF |  | REF |  | REF |  | REF |  | REF |  | REF |  | REF |  |
| Positive | 0.67 | 0.27-1.63 | 0.71 | 0.31-1.63 | 0.86 | 0.30-2.47 | 1.30 | 0.59-2.88 | 1.76 | 0.79-3.92 | 1.54 | 0.70-3.41 | 0.77 | 0.33-1.79 | 0.92 | 0.39-2.17 |
